# Revision of the genus *Heterosmylus* Krüger, 1913 from China (Neuroptera, Osmylidae)

**DOI:** 10.3897/zookeys.637.10310

**Published:** 2016-12-02

**Authors:** Min Dong, Han Xu, Yongjie Wang, Chunfeng Jia, Zhiqi Liu

**Affiliations:** 1 Department of Entomology, China Agricultural University, Beijing 100094, China; 2 College of Life Sciences, Capital Normal University, Beijing 100048, China

**Keywords:** Heterosmylus, Oriental region, Osmylidae, Palaearctic

## Abstract

A new species of osmylid (*Heterosmylus processus* **sp. n.**) is described and the other species in the genus from mainland China are redescribed. *Heterosmylus zhamanus* Yang, 1987, **syn. n.** is identified as a junior synonym of *Heterosmylus yunnanus* Yang, 1986. A key is provided to differentiate Palaearctic and Oriental species of *Heterosmylus*.

## Introduction

*Heterosmylus* Krüger is a relatively small genus of lance lacewing (Osmylidae: Protosmylinae) described by Krüger (1913a) from the Oriental Region. There are presently nine species in the genus, including seven species in mainland China, one species in Taiwan (*Heterosmylus primus* Nakahara) and another species (*Heterosmylus aspersus* Krüger) in northern India. Krüger (1913a) established the genus just based on the comparison with other genera, but the detailed description of the type species *Heterosmylus aspersus* was given in a subsequent publication (Krüger 1913b). As for the Chinese species, Nakahara (1955) described *Heterosmylus primus* from Taiwan. Subsequently, in a series of publications, Yang (1986, 1987, 1992, 1997, 1999) described an additional six species from mainland China. However, all the early taxonomists ignored or poorly described the characters of genitalia in osmylids, which result in a vague definition of the genus.

Herein the genus *Heterosmylus* is revised with a focus on the species from mainland China, and detailed descriptions of genitalic structures are provided for the first time. A revised diagnosis of the genus is proposed based on both external morphology and genitalic characters. The new species, *Heterosmylus processus* sp. n., is described and *Heterosmylus zhamanus* Yang, 1987, syn. n. is identified as a junior synonym of *Heterosmylus yunnanus* Yang, 1986. The distribution of the genus in China is also discussed.

## Material and methods

All the described specimens are deposited in the Entomological Museum of China Agricultural University
(CAU), Beijing. Terminalia preparations were made by macerating the apex of the abdomen in hot 10% KOH for 3–5 min, neutralized with 10% acetic acid. The apex of the abdomen was then transferred to glycerol for further dissection and examination. After examination it was moved to fresh glycerol and stored in a microvial pinned below the specimen. Images of wings were taken with a Nikon D7000 digital camera. Drawings were made under a light microscope. The terminology for wing venation and genitalia follows Winterton and Wang (2016).

## Taxonomy

### Heterosmylus

Taxon classificationAnimaliaNeuropteraOsmylidae

Krüger, 1913a

#### Type species.

*Heterosmylus aspersus* Krüger 1913a: 37, original designation. Deposited in Stettiner Museum (National Museum Szczecin, Poland).

#### Type locality.

India: Sikkim.

#### Diagnosis.

Head brown or dark brown; compound eyes black; antennae shorter than half length of forewing; prothorax black and length longer than width, with yellow setae; meso- and metathorax dark brown with long setae; legs yellow with short brown setae; forewings oblong and subacute at apex, with few brown spots; nygmata clear surrounded by light brown spot; veins thickened; costal cross-veins simple and occasionally bifurcate; forewing Rs with 8–15 branches, distal to the base of wing; cross-veins among Rs branches forming two or three series of gradates; forewing M branching more basally than the divergence of basal branch of Rs; no more than four cross-veins present between the two branches of M; forewing Cu bifurcating near the base of wing; CuA and CuP with numerous pectinate branches; CuP longer than half length of CuA; hind wings similar to forewings in size and shape without any spot apart from pterostigma; hindwing M branching near the base of wing; hindwing CuA with numerous pectinate branches; CuP simple and shorter than half length of CuA; male genitalia with 9^th^ tergite narrow, and sternite approximately quadrate; ectoproct relatively large, callus cerci rounded and located at the middle or underside of ectoproct; male genitalia composed of gonarcus, entoprocesses and mediuncus, arched gonarcus similar to other genera of Protosmylinae; mediuncus attached with a membrane below gonarcus, mediuncus bent into C-shape laterally; female genitalia with 9^th^ tergite narrow; spermathecae bent into n-shape with base expanded and apex columniform.

#### Included species.

*Heterosmylus aspersus* Krüger, *Heterosmylus curvagradatus* Yang, *Heterosmylus flavidus* Yang, *Heterosmylus limulus* Yang, *Heterosmylus primus* Nakahara, *Heterosmylus processus* sp. n., *Heterosmylus shennonganus* Yang, *Heterosmylus wolonganus* Yang, *Heterosmylus yunnanus* Yang.

#### Comments.

Although the type species of *Heterosmylus* was not described in detail when the genus was established, Krüger provided a detailed description of the type species in a following paper later that year (Krüger 1913a, b). This subsequent work was overlooked by Nakahara (1955), and later Ghosh (2000) presented a brief description for the species without the genitalic characters. Consequently, the systematic status of *Heterosmylus* was not well defined, although it is clear that the monophyly of *Heterosmylus* is well supported in the recent phylogenetic work on Osmylidae by Winterton et al. (in press). *Heterosmylus* also can be distinguished from other genera (*Gryposmylus* Krüger, *Lysmus* Navás, *Paryphosmylus* Krüger) in Protosmylinae based mainly on wing venation. In *Heterosmylus*, the veins are thickened and branches of forewing M have no more than four *ma-mp* cross-veins. In both *Gryposmylus* and *Lysmus* these are slender and the M vein generally five *ma-mp* cross-veins. Moreover, the base of costal-field of forewing of *Heterosmylus* species is narrower compared with that of *Gryposmylus*. *Heterosmylus* differs from *Paryphosmylus* in that the wings are mostly hyaline and single cross-vein presents before the separation of basal branch of Rs (Martins et al. 2016).

**Figure 1.**
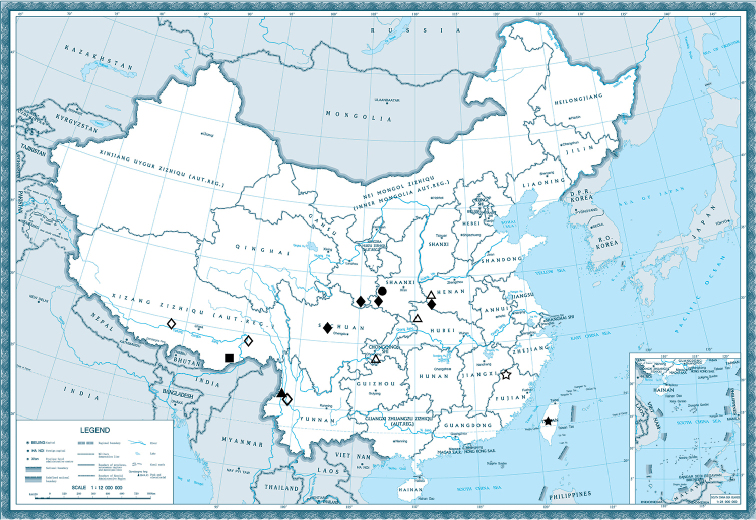
Distribution of *Heterosmylus* in China. ☆ = *Heterosmylus curvagradatus* ▲ = *Heterosmylus flavidus* ■ = *Heterosmylus limulus* ★ = *Heterosmylus primus* ● = *Heterosmylus processus* sp. n. △ = *Heterosmylus shennonganus* ♦ = *Heterosmylus wolonganus* ◇ = *Heterosmylus yunnanus*.

*Heterosmylus* is mainly recorded in the Oriental Region and especially in China (Fig. 1), typically in warmer and humid environments. According to the distribution of the individual species, we find *Heterosmylus wolonganus*, *Heterosmylus shennonganus* and *Heterosmylus yunnanus* with relatively wide distributions. It seems that *Heterosmylus wolonganus* has a broad geographical distribution from central to western China, occurring in four geographically continuous provinces, Sichuan, Gansu, Shaanxi and Henan. Interestingly, most localities of *Heterosmylus wolonganus* are along the boundary of Oriental and Palaearctic regions in China. *Heterosmylus shennonganus* is principally distributed in central China, representing a typical Oriental species. *Heterosmylus yunnanus* is another widespread species, distributed in Tibet, Yunnan and Sichuan. Considering the similar environment in these localities, it is estimated that these species might be present in the whole southwest of China. The other five species, *Heterosmylus curvagradatus*, *Heterosmylus limulus*, *Heterosmylus flavidus*, *Heterosmylus primus* and *Heterosmylus processus* sp. n., are only recorded in a single region. *Heterosmylus curvagradatus* is restricted to Fujian, while *Heterosmylus flavidus* is a distinctive species restricted in the west of Yunnan. *Heterosmylus limulus* is limited to Yadong (Tibet) and it could be found at the same altitude as *Heterosmylus yunnanus*. *Heterosmylus primus* is only recorded in Taiwan. We did not examine this species so we could not compare it with the other species, but *Heterosmylus curvagradatus* shows the similar appearance with this species, suggesting their potential close relationship. The new species *Heterosmylus processus* sp. n. is highly distinctive (weakly defined pterostigma and hyaline and colourless membrane) is found in Shaanxi near the Qinling Mountains.

### Key to *Heterosmylus* species (males) in the Palaearctic and Oriental regions

**Table:** 

1	Head and thorax with spots or stripes	**2**
–	Head and thorax without any spot and stripe; pronotum yellowish brown without any stripe, meso- and metanotum dark brown; cross-veins *rs-ma* with lance-shaped brown marks	***Heterosmylus aspersus***
2	Mediuncus with a process at base (Fig. 4); membrane of wings hyaline and veins not edged with spots (Fig. 2); pronotum with four brown spots along anterior margin, two round brown spots in middle; mesonotum with two brown spots in middle on both sides	***Heterosmylus processus* sp. n.**
–	Mediuncus without any process at base, membrane of wings only with a few spots	**3**
3	Forewings with many spots including along the gradate cross-veins	**4**
–	Forewings with few spots in the membrane (Fig. 21); pronotum with two yellow narrow stripes in middle; metanotum with two brown spots on anterior margin	***Heterosmylus wolonganus***
4	More than 11 branches of Rs	**5**
–	11 or fewer branches of Rs	**7**
5	An oblique brown stripe present from pterostigma to the outer margin, Cu with four brown spots; pronotum with two pale yellow longitudinal stripes, mesonotum brown anteriorly	***Heterosmylus limulus***
–	No oblique stripes present from pterostigma to the outer margin	**6**
6	Pronotum black with a black longitudinal stripe medially; meso- and metanotum black; Rs with 14–17 branches; Cu edged with 3–4 yellow spots; apex of gonarcus bent upwards and relative long in lateral view	***Heterosmylus yunnanus***
–	Pronotum brown with a yellowish longitudinal stripe medially, meso- and metanotum brown; vertex with a Y-shaped mark	***Heterosmylus primus***
7	Membrane of wings yellow and gradate cross-veins not edged with spots (Fig. 29); pronotum with a dark brown longitudinal stripe medially	***Heterosmylus flavidus***
–	Membrane of wings hyaline and gradate cross-veins edged with brown	**8**
8	Apex of mediuncus short, broad and flat in dorsal view (Figs 46–47); ectoproct without any process in lateral view; two yellow longitudinal stripes present from pronotum to mesonotum; mesoscutellum bright yellow, metanotum with a central yellow stripe	***Heterosmylus curvagradatus***
–	Apex of mediuncus long, acute and protuberant in dorsal view (Figs 38–39); ectoproct with a dorsal coniform process in lateral view; pronotum with two narrow longitudinal dark brown marks medially	***Heterosmylus shennonganus***

### Heterosmylus processus sp. n.

Taxon classificationAnimaliaNeuropteraOsmylidae

http://zoobank.org/18F14A57-CA9C-4329-BA1F-F885461CCCB6

Figs 2 , 3–6

#### Material examined.

Holotype Male. CHINA: Shaanxi (Province): Taibai, [33°55'N, 107°43'E] 09.v.1982, leg. Guojun Qi. [Verbatim label data translated from Chinese]: CHINA: Shaanxi, Taibai/ 09.v.1982/ Guojun Qi/ CAU. Terminalia cleared in KOH, and stored in a micro-vial pinned below the specimen.

#### Diagnosis.

Pronotum with four brown spots at anterior margin, two round brown spots in middle and two brown spots at posterior margin; membrane hyaline, pterostigma yellow, without dark spots besides the pterostigma; veins light yellow at base but brown from middle to the end; mediuncus C-shaped in lateral view with a basal process and boat-shaped in dorsal view.

#### Description.

**Figure 2.**
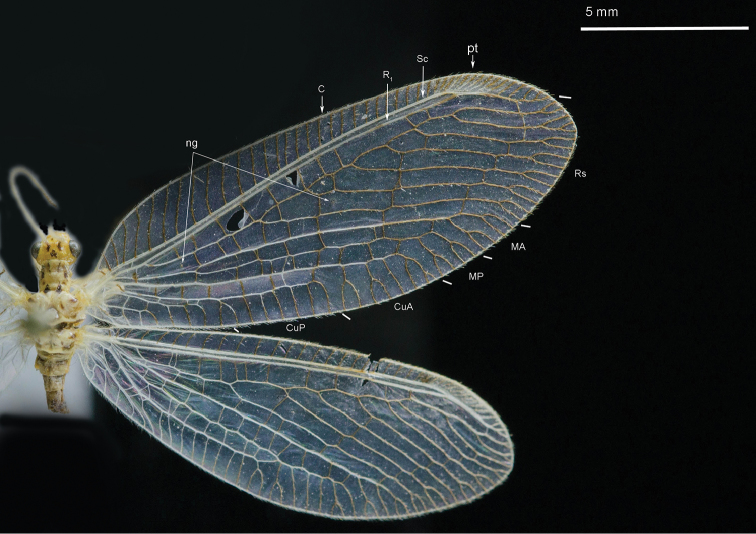
Wings of *Heterosmylus processus* sp. n. Abbreviations: ng, nygmata; pt, pterostigma.

Body length 8.6 mm. *Head*. Vertex yellow with brown setae; ocelli distinctively brown; compound eyes black; antennae yellow with a brown stripe at base; frons with two brown stripes, genae with two round brown spots; maxillary palpi yellow and thick, labium short and brown. *Thorax*. Yellow dorsally and dark brown ventrally; pronotum with four brown spots at anterior margin, two round brown spots in middle and two brown spots at posterior margin linking to the mesonotum; mesonotum with two brown spots in middle on both sides; metanotum similar to the mesonotum, with two spots in middle. *Legs*. Yellow with brown setae; claws brown with a small tooth. *Wings* (Fig. 2): Forewing length 15.4 mm, width 6.2 mm; membrane hyaline without any spot besides the yellow pterostigma; veins yellow; costal field broad, cross-veins simple without forks. Cross-veins in radial sector few besides the gradate cross-veins. Rs with 8 branches. The basal cross-vein between R_1_ and M edged with a brown mark. Hind wing length 14.4 mm, width 5.3 mm; membrane hyaline, veins light yellow at base but brown from middle to the end. Costal field narrow, pterostigma yellow, and Rs with 7 branches. *Abdomen*. Yellow dorsally, dark brown ventrally, covered with yellow setae.

**Figures 3–6.**
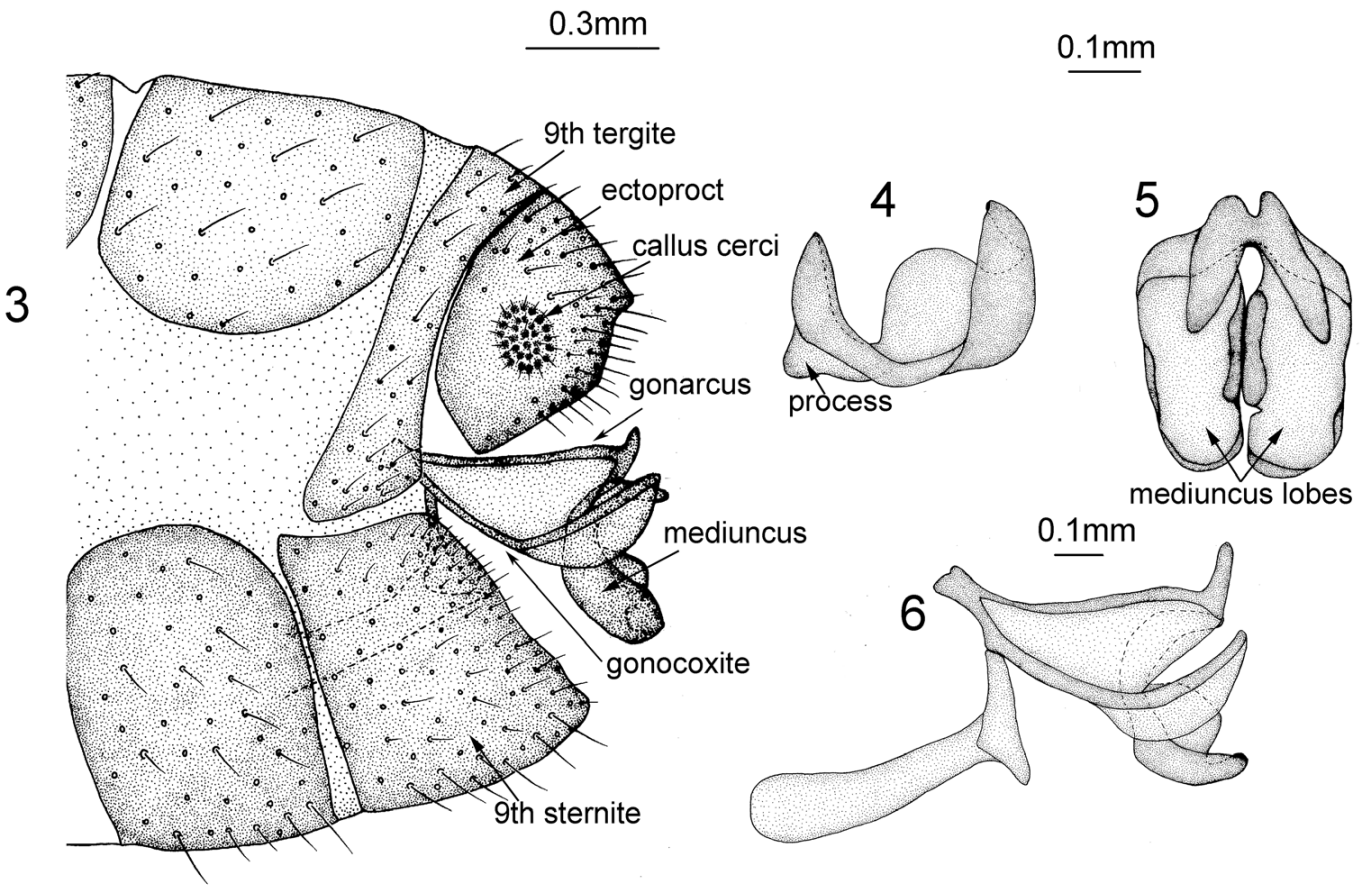
*Heterosmylus processus* sp. n. Male: **3** terminalia, lateral view **4** mediuncus, lateral view **5** mediuncus, dorsal view **6** genitalia, lateral view.

*Male Terminalia* (Figs 3–6). Ectoproct quadrate in lateral view; callus cerci rounded and small. Distal part of gonarcus bent upwards. Entoprocesses curved in middle and dilated apically. Mediuncus (Figs 4–5) C-shaped in lateral view with a basal process and boat-shaped in dorsal view; two mediuncus lobes fused at base and each one raised on both sides, the inner side larger than the outer one.

#### Distribution.

China (Shaanxi).

#### Etymology.

The specific name refers to the process at the base of the mediuncus.

#### Remarks.

This species is known from its type locality, Shaanxi province. It is easily distinguished from other species in the genus because of the body coloration, morphology and the genital characters. *Heterosmylus processus* sp. n. has a smaller body size and hyaline wings, while the others usually have a patterned membrane. Moreover, the mediuncus within the new species possesses a distinct process at the base whereas it is absent in the other species of *Heterosmylus*.

### Heterosmylus yunnanus

Taxon classificationAnimaliaNeuropteraOsmylidae

Yang, 1986

Figs 7 , 8–13

Heterosmylus zhamanus Yang, 1988: 195. **syn. n.**

#### Material examined.

Holotype male. CHINA: Yunnan (Province): Lushuixian Gangfang, [25°57'N, 98°52'E], 29.ix.1980, leg. Dejing Zou. [Verbatim label data (translated from Chinese)]: CHINA: Yunnan, Lushuixian, Gangfang, 29.ix.1980/ Dejing Zou. Terminalia cleared in KOH, and stored in a micro-vial pinned below the specimen. 3 males, 1 female (type specimens of *Heterosmylus zhamanus*), Tibet: Zhangmu, 06.vi.1981, leg. Shengchang Hu; 3 males, 5 females, Tibet: Hanmi, 24.viii.2005, leg. Dakang Zhou; 2 males, Sichuan: Luding, Hailuogou, 26.vi.2006, leg. Xiaoshuan Bai.

#### Diagnosis.

Pronotum black with two yellowish longitudinal stripes at anterior margin; mesonotum with two yellow spots in middle; apex of gonarcus bent upwards and relative long in lateral view.

#### Redescription.

**Figure 7.**
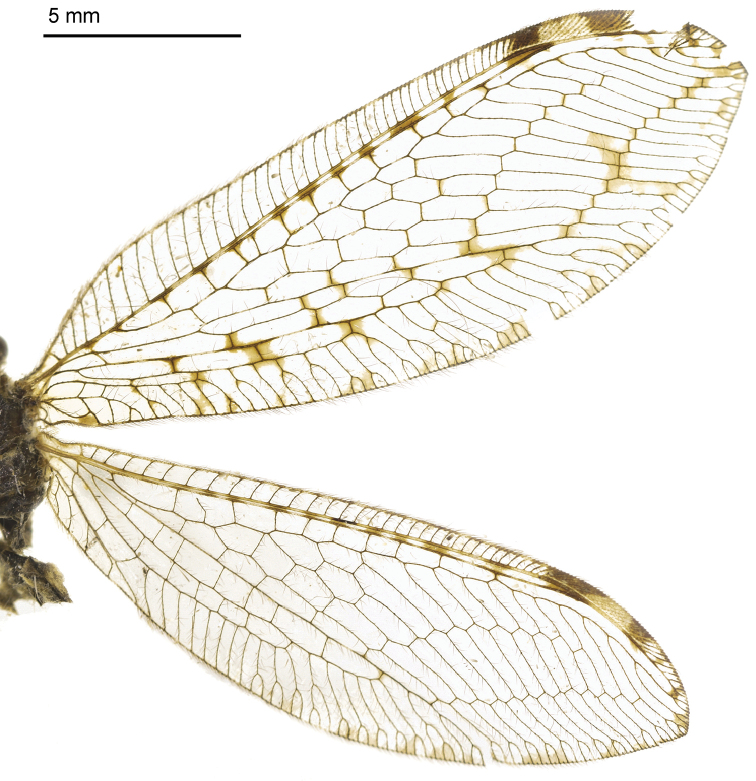
Wings of *Heterosmylus yunnanus* Yang, 1986.

Body length 9–11 mm. *Head*. Vertex dark brown, frons bright yellow; ocelli yellow; compound eyes dark grey; antennae entirely dark; clypeus brownish, maxillary and labial palpi dark brown. *Thorax*. Pronotum black with two yellowish longitudinal stripes at anterior margin; mesonotum with two yellow spots in middle. *Wings* (Fig. 7). Forewings length 18–21 mm, width 6–7 mm; membrane hyaline, and veins dark brown; pterostigma dark brown with yellowish center. Rs with 15–17 branches; Cu edged with three or four yellow spots. Hind wing length 15–18 mm, width 5–6 mm; membrane hyaline, cross-veins edged with brown marks between Sc and R_1_.

**Figures 8–13.**
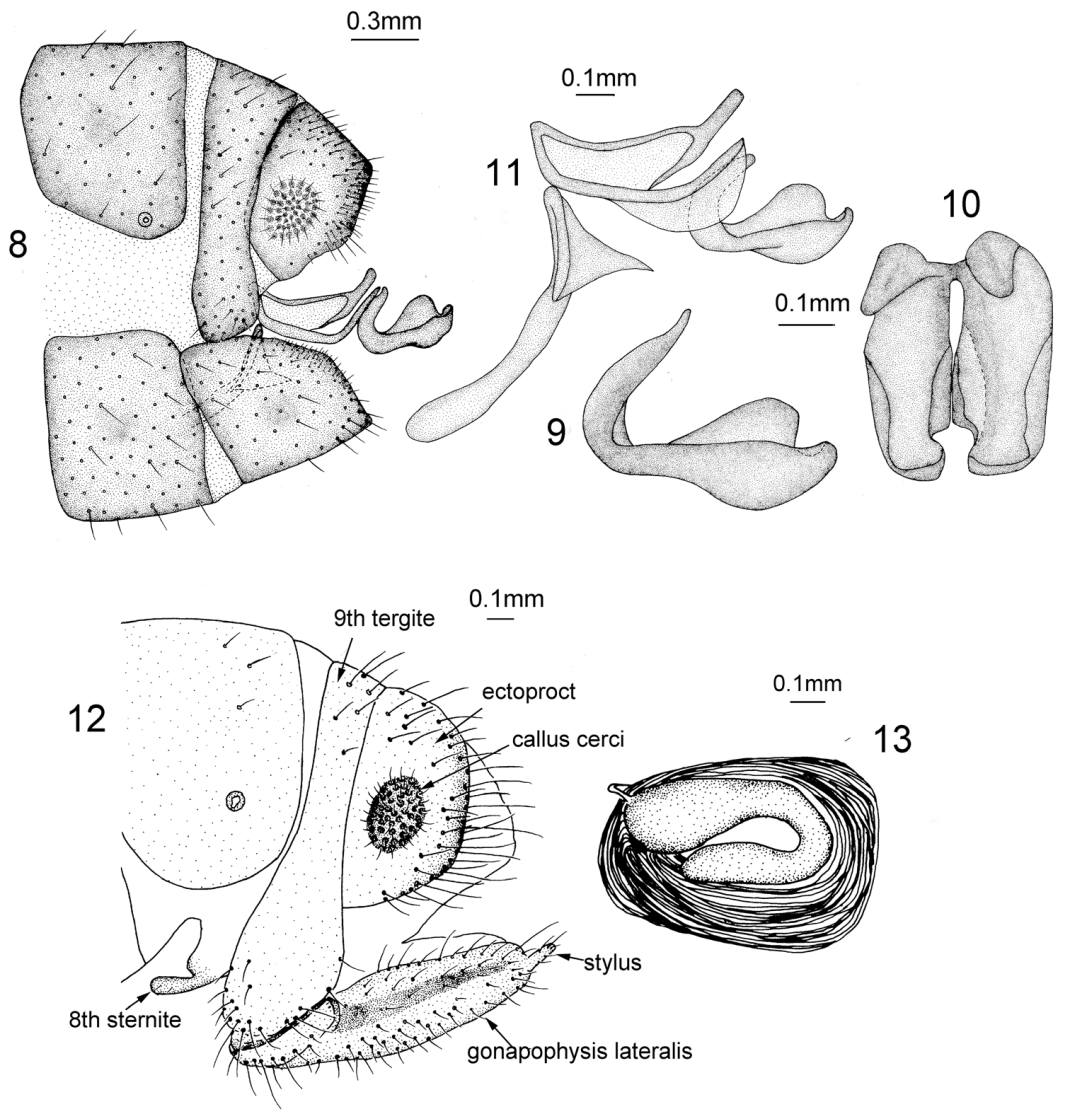
*Heterosmylus yunnanus* Yang, 1986. Male: **8** terminalia, lateral view **9** mediuncus, lateral view **10** mediuncus, dorsal view **11** genitalia, lateral view Female: **12** terminalia, lateral view **13** spermatheca, lateral view.

*Male Terminalia* (Figs 8–11). Ectoproct quadrate in lateral view. Gonarcus rod-like in lateral view, the apex bent upwards and relatively long; entoprocesses distally expanded, lobe-shaped; mediuncus fused at base and curved into C-shape in lateral view; each mediuncus lobe raised on both sides, boat-shaped in dorsal view.

*Female Terminalia* (Figs 12–13). Ectoproct trapeziform in lateral view; 9^th^ gonocoxite finger-shaped in lateral view, 9^th^ gonostylus brown and long. Spermathecae bent into C-shaped and basal part longer than distal.

#### Distribution.

China (Tibet, Yunnan, Sichuan)

#### Remarks.

Although the original identification of both *Heterosmylus yunnanus* Yang and *Heterosmylus zhamanus* Yang was based on the colour pattern of the head, there are no convincing differences to distinguish them after comparison of their genitalic features. So we believe that *Heterosmylus zhamanus* should be a synonym of *Heterosmylus yunnanus*.

### Heterosmylus limulus

Taxon classificationAnimaliaNeuropteraOsmylidae

Yang, 1987

Figs 14 , 15–20

#### Material examined.

Holotype Male, CHINA: Tibet: Yadong, [27°31'N, 88°55'E], 24.viii.1978, leg. Fasheng Li. [Verbatim label data (translated from Chinese)]: CHINA: Tibet, Yadong/ 24.viii.1978/ Fasheng Li/ CAU. 1 male, same data as Holotype. 1 female, China: Tibet: Yadong, 30.viii.1984, leg. Yongxiang Zhao.

#### Diagnosis.

Pronotum with two yellow stripes in middle, an oblique brown mark presenting from the pterostigma to the posterior outer margin of forewing; base of mediuncus slender and apex dilated as lobe-shape in lateral view.

#### Redescription.

**Figure 14.**
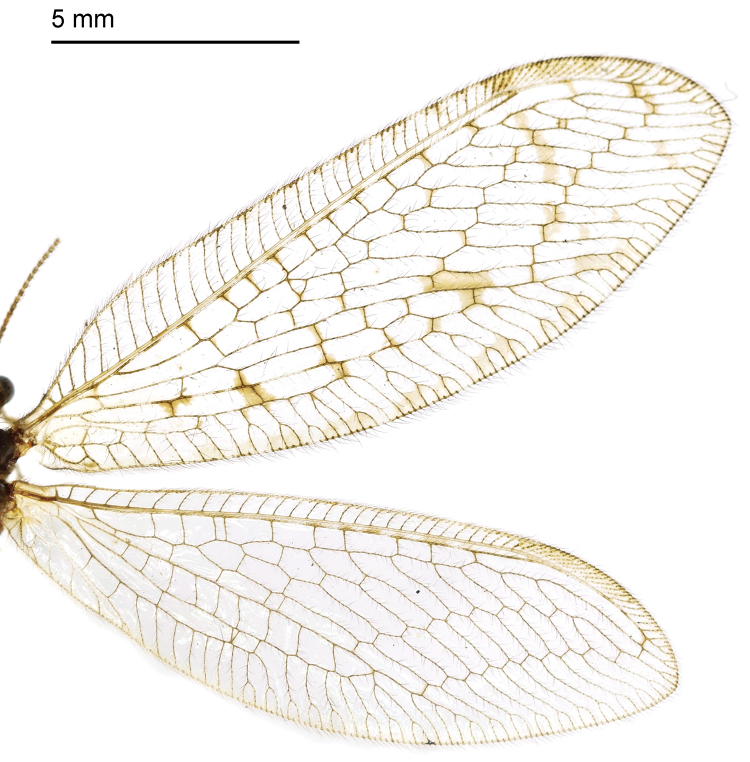
Wings of *Heterosmylus limulus* Yang, 1987.

Body length 8.0 mm. *Head*. Vertex dark brown, frons brown. Ocelli yellowish, eyes dark grey. Antennae brown, encircled by a yellowish stripe at base. Clypeus yellow, maxillary palpi and labial palpi dark brown. *Thorax*. Dark brown. Pronotum with two light yellow stripes medially; mesonotum brown at anterior part with black setae. *Wings* (Fig. 14). Forewing length 16–17 mm, width 5–6 mm; membrane hyaline, veins brown; pterostigma yellow, but brown on both sides; an oblique brown stripe presenting from the pterostigma to the posterior outer margin; Cu with four brown spots. Outer gradate series of cross-veins and wing margin bordered with fuscous spots. Rs with 13–15 branches. Hind wing length 13–14 mm, width 4–5 mm; membrane hyaline with some light brown spots between Sc and R_1_.

**Figures 15–20.**
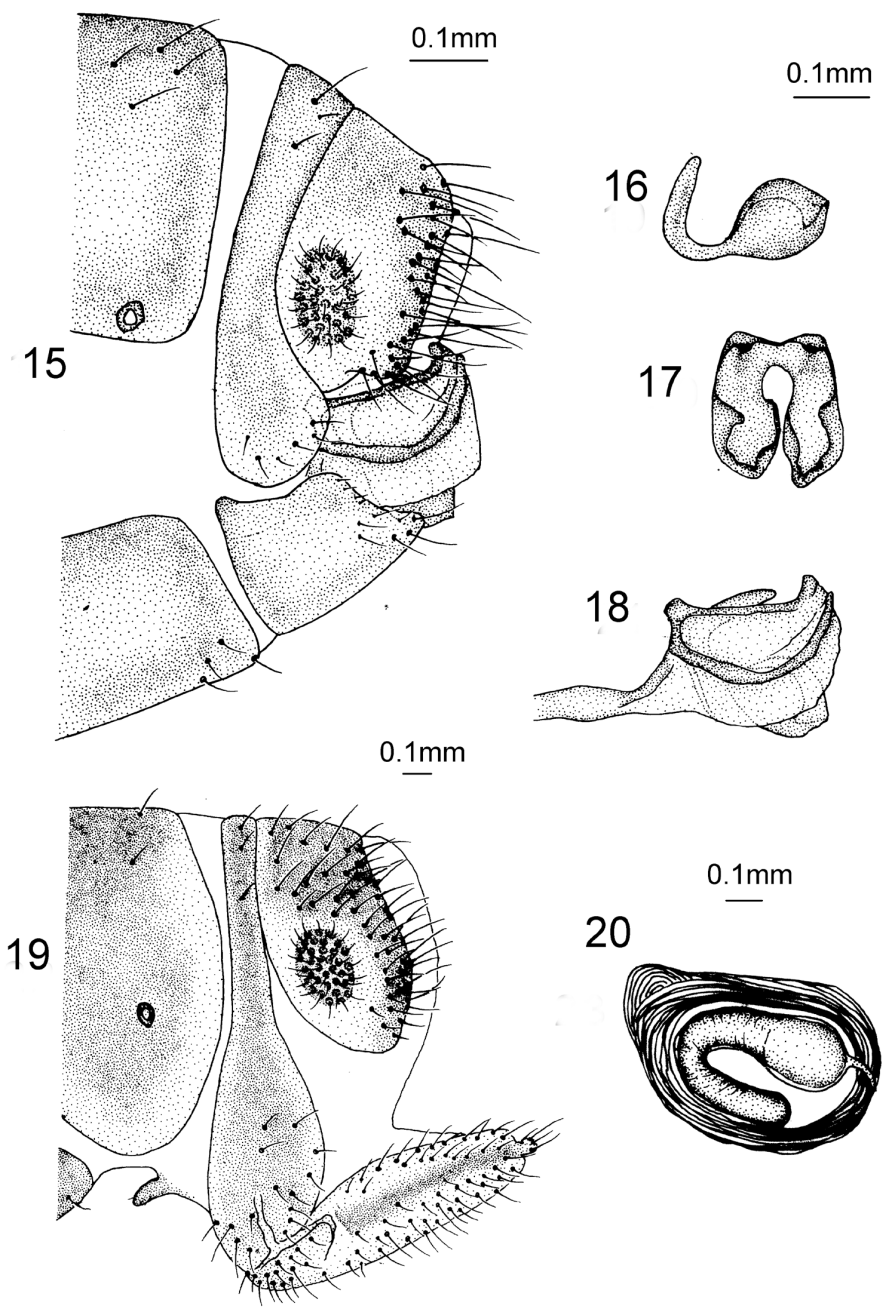
*Heterosmylus limulus* Yang, 1987. Male: **15** terminalia, lateral view **16** mediuncus, lateral view **17** mediuncus, dorsal view **18** genitalia, lateral view. Female: **19** terminalia, lateral view **20** spermatheca, lateral view.

*Male Terminalia* (Figs 15–18). Ectoproct quadrate in lateral view; gonarcus rod-like in lateral view, distal part upswept and relatively small; mediuncus fused at base and curved into C-shape in lateral view; the base slender and apex dilated as lobe-shape in lateral view.

*Female Terminalia* (Figs 19–20). Ectoproct approximately oblong in lateral view; 9^th^ gonocoxite fusiform in lateral view; 9^th^ gonostylus brown and small; spermathecae bent into C-shape.

#### Distribution.

China (Tibet).

### Heterosmylus wolonganus

Taxon classificationAnimaliaNeuropteraOsmylidae

Yang, 1992

Figs 21 , 22–28

#### Material examined.

Holotype Male. CHINA: Sichuan (Province): Wolong, [31°01'N, 103°10'E], 25.vii.1993, leg. Shuyong Wang. [Verbatim label data (translated from Chinese)]: CHINA: Sichuan Prov., Wonglong/ 25.vii.1993/ Shuyong Wang/ CAU. 1 male, CHINA: Shaanxi (Province): Ningshan, 18.vi.1981, leg. Chikun Yang. 1 female, CHINA: Shaanxi (Province): Huangniupu, 15.viii.1981, leg. Weidong Wang. 1 male, CHINA: Shaanxi (Province): Taibaishan, 09.iii.1982, leg. Chikun Yang. 1 male, CHINA: Shaanxi (Province): Taibaishan, 09.v.1982, leg. Guojun Qi. 1 female, CHINA: Shaanxi (Province): Taibaishan, 15.vii.1982, leg. Jingruo Zhou, Lan Liu. 1 male, 1 female, CHINA: Shaanxi (Province): Ningshan, 18.vii.1982, leg. Shenghui Lei. 1 female, CHINA: Shaanxi (Province): Ningshan, Xiangyang, Fengqi, 18.vii.1982, leg. Deqing Wang. 2 males, CHINA: Shaanxi (Province): Nanzheng, Yuanba, 27.v.1983, leg. Dahan He. 1 male, CHINA: Gansu (Province): Wenxian, Bikou, 25.vii.1998, leg. Jun Chen. 2 males, CHINA: Henan (Province): Songxian, Baiyunshan, 19.vii.1996, leg. Xiaocheng Shen. 10 males, 12 females, CHINA: Henan (Province): Songxian, Baiyunshan, 14-18.vii.2004, leg. Bingzhen Yan.

#### Diagnosis.

Pronotum with two yellow narrow stripes in middle; metanotum with two brown spots on anterior margin; gonarcus sclerotized distally and bent upward and hook-shaped in lateral view; base of mediuncus approximately finger-shaped in lateral view.

#### Redescription.

Body length 9–11 mm. *Head*. Vertex yellowish brown; frons yellow with one dark brown spot near antennae; ocelli brown, eyes blackish brown; antennae blackish brown; clypeus yellow, maxillary and labial palpi dark brown

**Figure 21.**
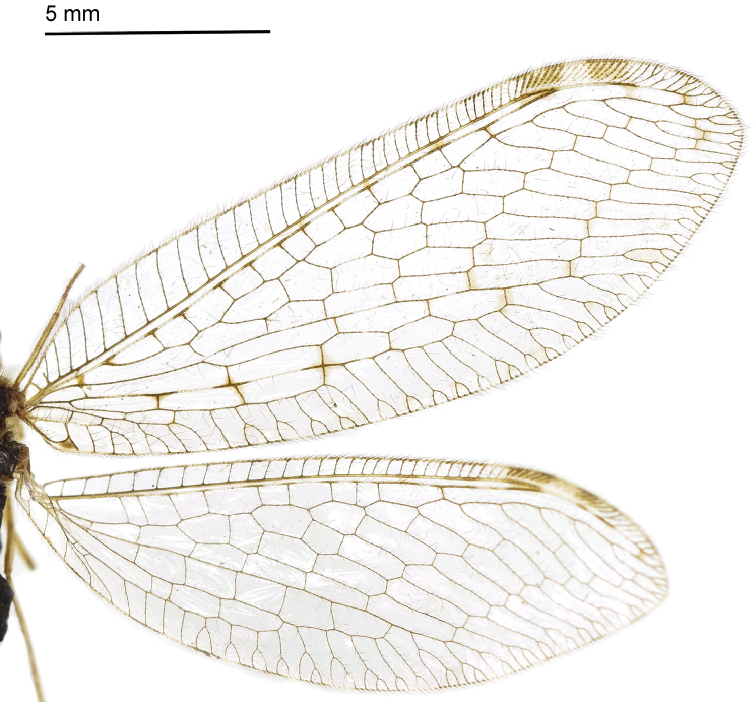
Wings of *Heterosmylus wolonganus* Yang, 1992.

*Thorax*. Pronotum with two yellow narrow stripes in middle with some dark setae on both sides; mesonotum with two yellow longitudinal spots; metanotum with two brown spots on anterior margin. *Wings* (Fig. 21). Forewing length 18–20 mm, width 6–7 mm; membrane hyaline, veins mostly brown; pterostigma light yellow but brown on both sides; Rs with 10 branches. Four cross-veins present between MA and MP. Hind wing length 16–17 mm, width 5–6 mm; Rs with 11–12 branches, without any distinct spot besides the pterostigma.

**Figures 22–28.**
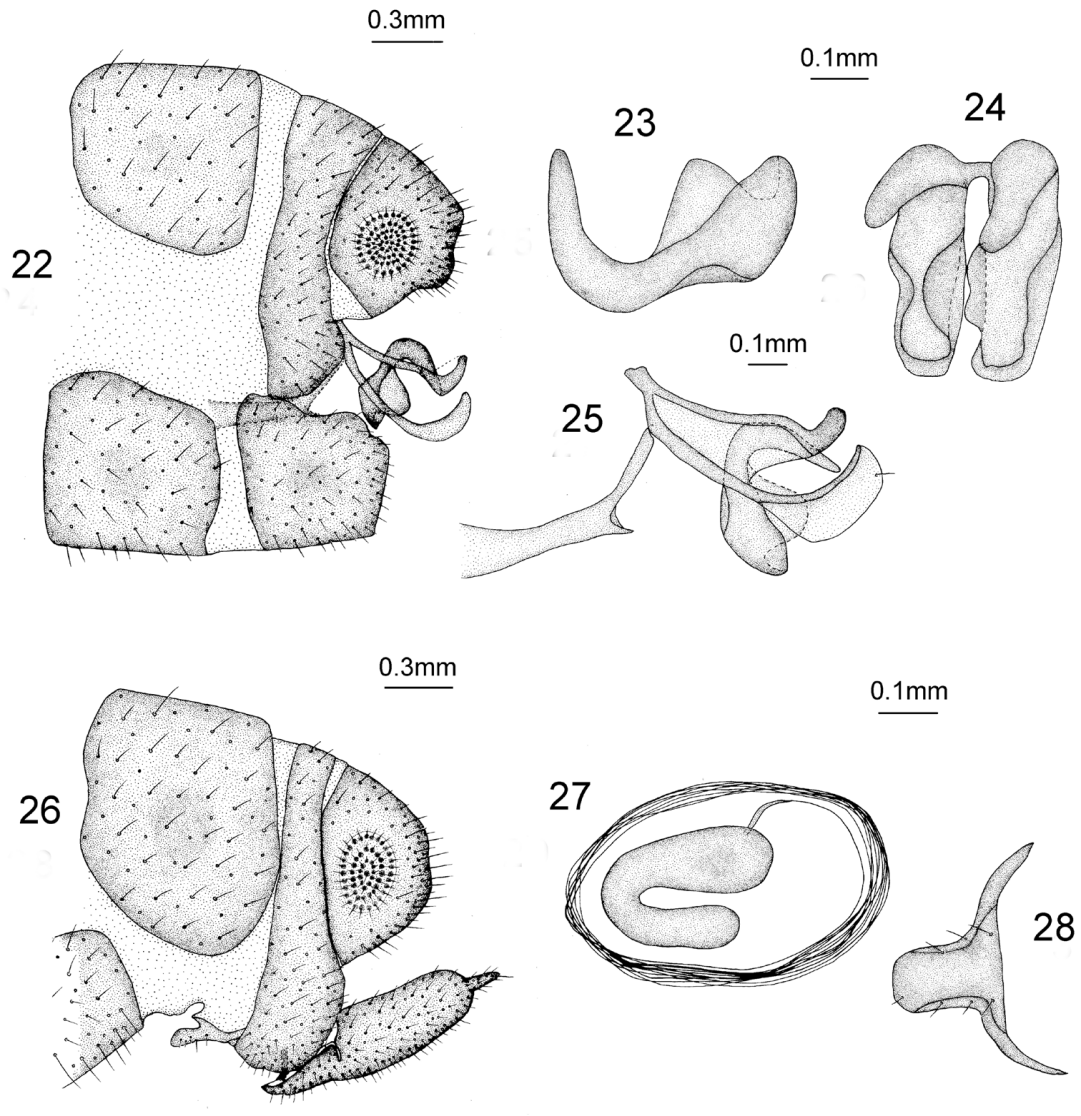
*Heterosmylus wolonganus* Yang, 1992. Male: **22** terminalia, lateral view **23** mediuncus, lateral view **24** mediuncus, dorsal view **25** genitalia, lateral view. Female: **26** terminalia, lateral view **27** spermatheca, lateral view **28** 8^th^ sternite, ventral view.

*Male Terminalia* (Figs 22–25). Ectoproct quadrate in lateral view; gonarcus sclerotized distally and bent upwards as hook in lateral view; gonocoxite bent and distally dilated as lobe-shaped in lateral view mediuncus curved into C-shape in lateral view and spoon-shaped in dorsal view; base approximately finger-shaped in lateral view.

*Female Terminalia* (Figs 26–28). Anterior part of 8^th^ sternite reduced and short finger-shaped, posterior part broad; ectoproct broad and trapezoid in lateral view; 9^th^ gonocoxite fusiform in lateral view; 9^th^ gonostylus long in lateral view; spermatheca bent into C-shape.

#### Distribution.

China (Sichuan, Shaanxi, Gansu, Henan).

### Heterosmylus flavidus

Taxon classificationAnimaliaNeuropteraOsmylidae

Yang, 1992

Figs 29 , 30–35

#### Material examined.

Holotype Female. CHINA: Yunnan (Province): Lushui, Yaojiapin, [25°58'N, 98°42'E], 04.vi.1981, leg. Shuyong Wang. [Verbatim label data (translated from Chinese)]: CHINA: Yunnan Prov., Lushui, Yaojiaping/ 04.vi.1981/ Shuyong Wang/ CAU. Paratype Male, CHINA: Yunnan (Province): Lushui, Pianma, 29.v.1981. leg. Xuezhong Zhang. [Verbatim label data (translated from Chinese)]: CHINA: Yunnan Prov., Lushui, Pianma/ 29.v.1981/ Xuezhong Zhang/ CAU.

#### Diagnosis.

Pronotum with a dark brown longitudinal stripe medially; metanotum with a brown spot in middle; forewing light yellow; mediuncus C-shaped in lateral view and scoop-shaped in dorsal view.

#### Redescription.

**Figure 29.**
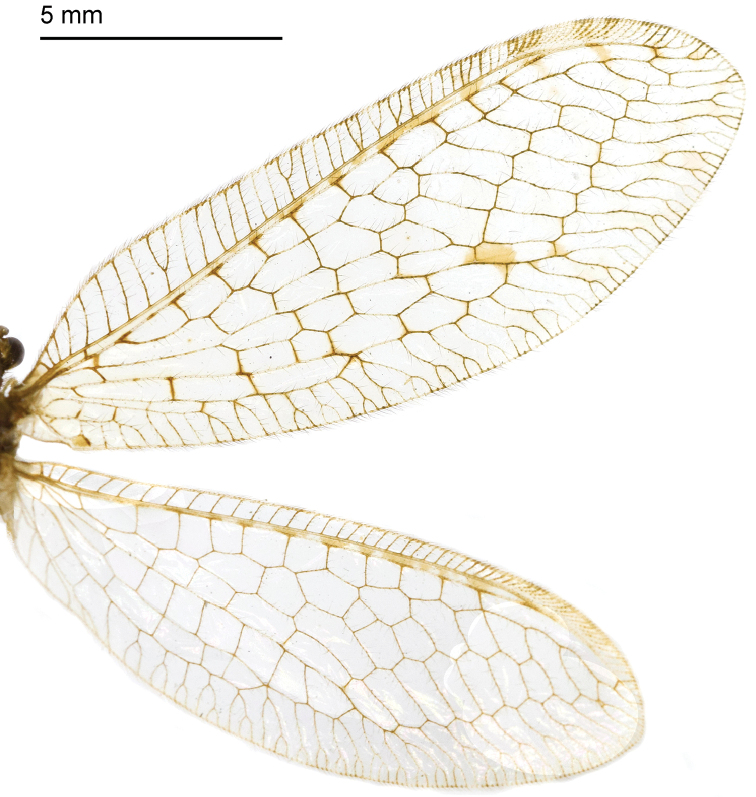
Wings of *Heterosmylus flavidus* Yang, 1992.

Body length 7–9 mm. *Head*. Vertex dark brown, frons yellow; ocelli yellowish; Compound eyes brown with some small spots; antennae fuscous except for the yellow scape; clypeus yellow, maxillary and labial palpi black. *Thorax*. Mostly yellow. Pronotum with a brown longitudinal stripe, and with some long setae on both sides; mesonotum brown on margin; metanotum dark brown with a brown spot medially. *Wings* (Fig. 29). Forewing length 16–17 mm, width 5–6 mm; membrane light yellow, veins brown and thickened; pterostigma light yellow but brown on both sides; R_1_ edged with some brown spots, Rs with 11 branches, cross-veins among branches of Rs forming three series of gradates. Hind wing length 12–14 mm, width 4–6 mm; membrane hyaline with small spots between Sc and R_1_.

**Figures 30–35.**
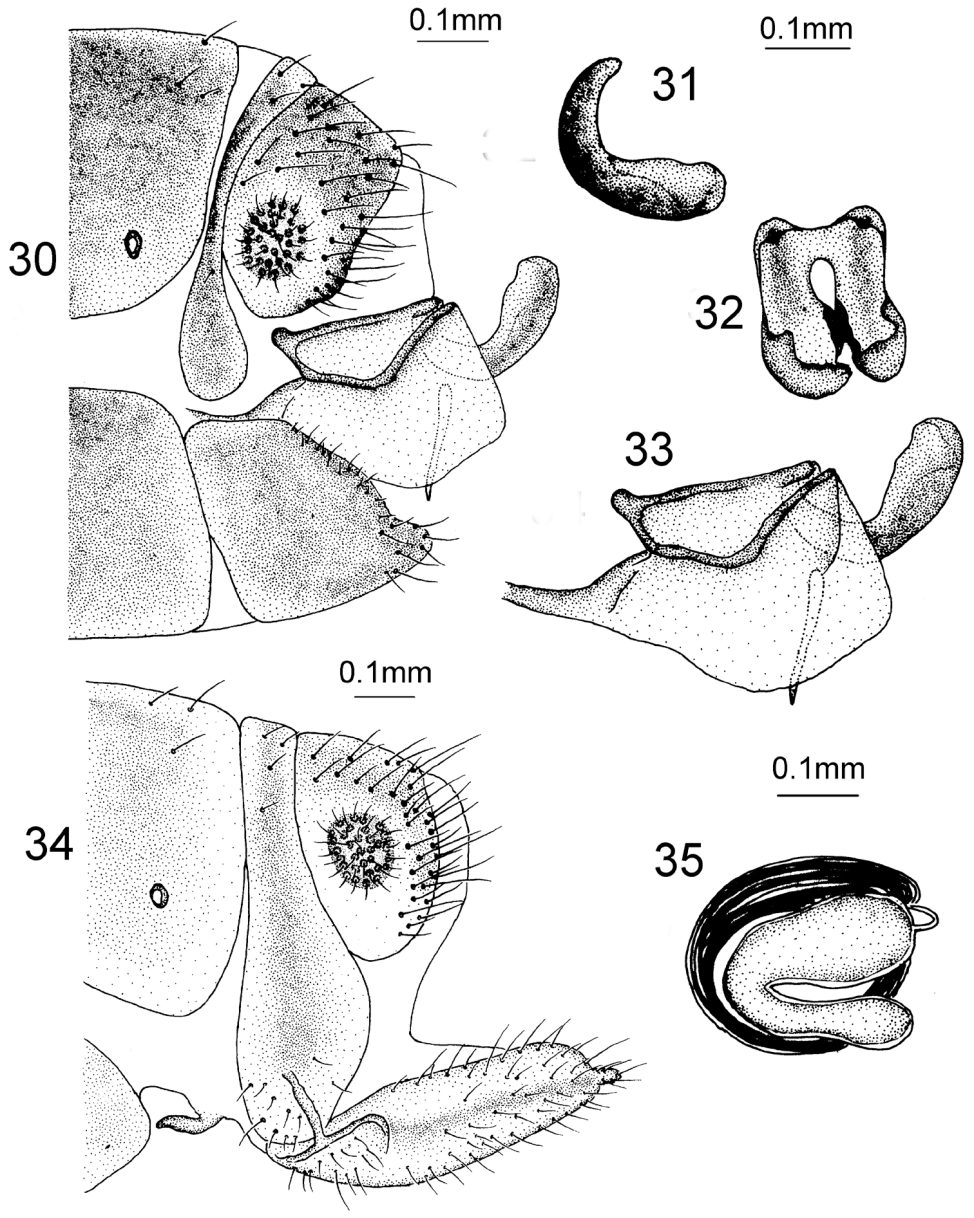
*Heterosmylus flavidus* Yang, 1992. Male. **30** terminalia, lateral view **31** mediuncus, lateral view **32** mediuncus, dorsal view **33** genitalia, lateral view. Female: **34** terminalia, lateral view **35** spermatheca, lateral view.

*Male Terminalia* (Figs 30–33). Ectoproct quadrate in lateral view; gonarcus rod-like, but apex not bent upwards in lateral view; gonocoxite bent in the middle; mediuncus C-shaped in lateral view and scoop-shaped in dorsal view.

*Female Terminalia* (Figs 34–35). 8^th^ sternite finger-shaped in lateral view; ectoproct broad and approximately elliptical in lateral view; 9^th^ gonocoxite finger-shaped in lateral view; 9^th^ gonostylus relatively long and finger-shaped in lateral view; spermathecae bent into C-shape, base thicker than apex.

#### Distribution.

China (Yunnan).

### Heterosmylus shennonganus

Taxon classificationAnimaliaNeuropteraOsmylidae

Yang, 1997

Figs 36 , 37–43

#### Material examined.

Holotype Male. CHINA: Hubei (Province): Shennongjia, [31°42'N, 110°38'E], 21.viii.1985, leg. Xiaoyuan Mao. [Verbatim label data (translated from Chinese)]: CHINA: Hubei Prov., Shennongjia, 21.viii.1985/ Xiaoyuan Mao/ CAU. Paratype 4 males, 3 females, same data as the holotype. 1 female, Henan: Luanchuan, Longyuwan, 1997.08.14, leg. RLS. 1 male, CHINA: Henan (Province): Luanchuan, Longyuwan, 18.viii.1997, leg. RLS. 1 male, CHINA: Henan (Province): Luanchuan, Longyuwan, 19.vii.2004, leg. Bingzhen Yan. 1 female, CHINA: Henan (Province): Neixiang, Baotianman, 23.vii.2004, leg. Bingzhen Yan. 1 male, CHINA: Henan (Province): Neixiang, Baotianman, 24.vii.2004, leg. Bingzhen Yan. 1 male, CHINA: Chongqing: Jiangjin, Simianshan, 21.ix.2007, leg. Weiwei Zhang. 1 female, CHINA: Shaanxi (Province): Xiangyangba, 24.viii.1982, leg. Shenghui Lei.

#### Diagnosis.

Pronotum with two narrow longitudinal dark brown marks in middle; ectoproct with a dorsal coniform process in lateral view; gonarcus with a short finger-like process distally in lateral view.

#### Redescription.

Body length 8–10 mm. *Head*. Vertex with a brown cross-stripe; frons yellow but brown on both sides; ocelli large and prominent, compound eyes shiny black; antennae dark brown; clypeus fulvous, maxillary and labial palpi brown.

**Figure 36.**
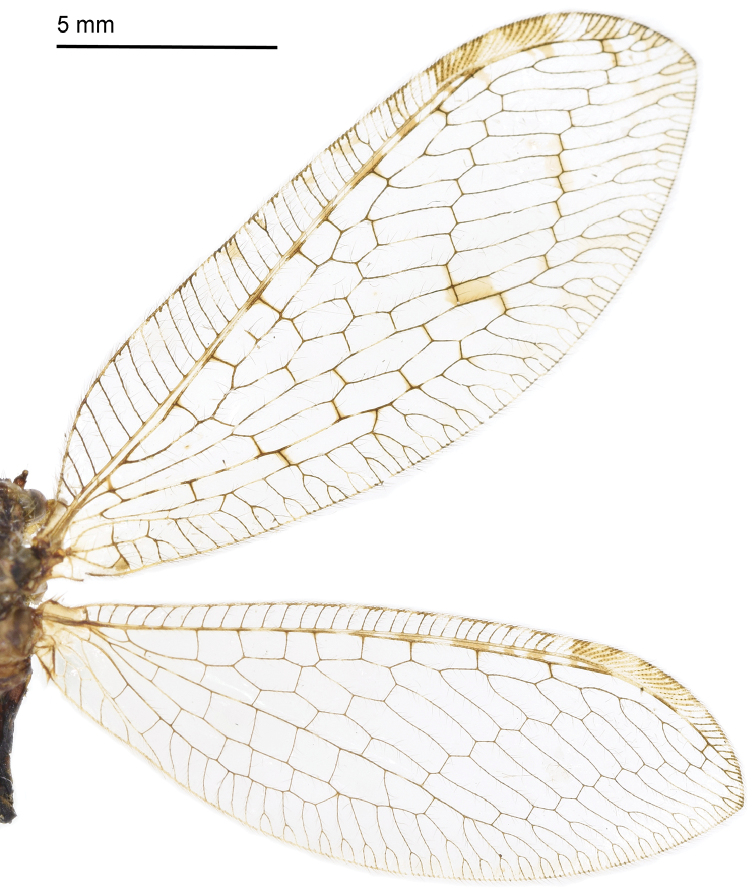
Wings of *Heterosmylus shennonganus* Yang, 1997.

*Thorax*. Pronotum with two narrow longitudinal dark brown marks in the middle; mesonotum with dark setae; metanotum without spots. *Wings* (Fig. 36). Forewing length 16–17 mm, width 6.4 mm; membrane hyaline, veins mainly brown; pterostigma yellowish but brown on both sides; costal field with three or four brown spots; *r1-rs* edged with brown marks; Rs with 8–9 branches, cross-veins among branches of Rs forming two series of gradates; the outer gradate cross-veins edged with brown spots. Hind wing length 15.5 mm, width 5.4 mm. Membrane hyaline with few spots.

**Figures 37–43.**
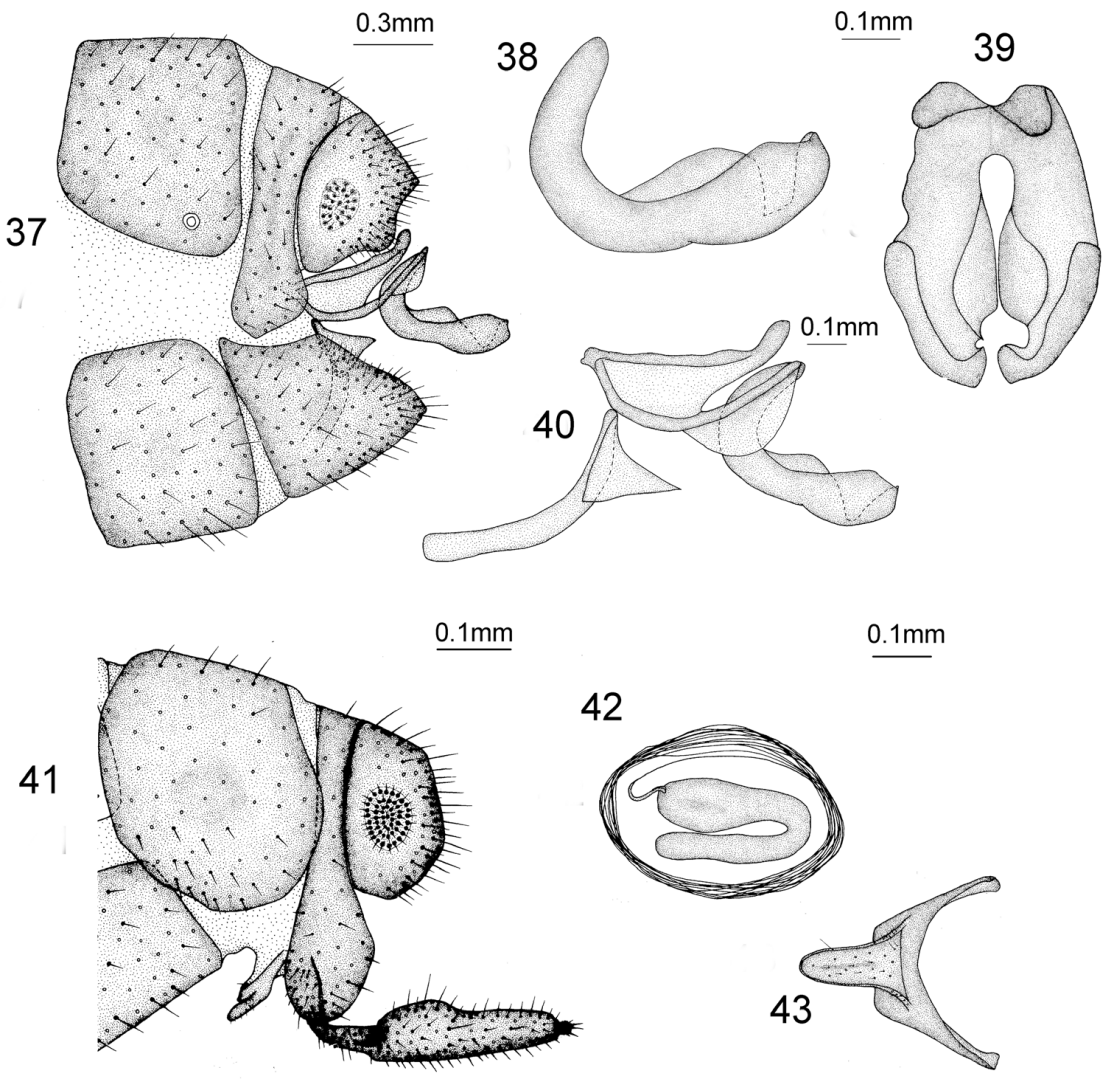
*Heterosmylus shennonganus* Yang, 1997. Male **37** terminalia, lateral view **38** mediuncus, lateral view **39** mediuncus, dorsal view **40** genitalia, lateral view. Female: **41** terminalia, lateral view **42** spermatheca, lateral view **43** 8^th^ sternite, ventral view.

*Male Terminalia* (Figs 37–40). Ectoproct approximately quadrate with a dorsal coniform process in lateral view; gonarcus approximately rod-like and distally sclerotized with a short finger-like process in lateral view; gonocoxite curved as ancon and distally dilated as lobe; mediuncus curved and thickened in lateral view and boat-shaped with a cone-shaped apex in dorsal view.

*Female Terminalia* (Figs 41–43). 8^th^ sternite approximately finger-shaped in lateral view. Ectoproct trapeziform in lateral view; 9^th^ gonocoxite approximately finger-shaped in lateral view; 9^th^ gonostylus finger-shaped and brown; spermathecae bend into C-shape.

#### Distribution.

China (Henan, Shaanxi, Hubei, Chongqing).

### Heterosmylus curvagradatus

Taxon classificationAnimaliaNeuropteraOsmylidae

Yang, 1999

Figs 44 , 45–50

#### Material examined.

Holotype Male. CHINA: Fujian (Province): Wuyishan, Huanghuacong, [27°48'N, 117°42'E], 13.x.1980, leg. Fan Jiang. [Verbatim label data (translated from Chinese)]: CHINA: Fujian Prov., Wuyishan, Huanghuacong/ 13.x.1980/ Fan Jiang/ CAU. Paratype Female. CHINA: Fujian (Province): Wuyishan, Xianfenling, 19.ix.1987. leg. Jiashe Wang.

#### Diagnosis.

Two yellow longitudinal stripes present from pronotum to mesonotum. mesoscutellum bright yellow, metanotum with a central yellow stripe; apex of gonarcus slightly dilated, short and curved dorsally.

#### Redescription.

**Figure 44.**
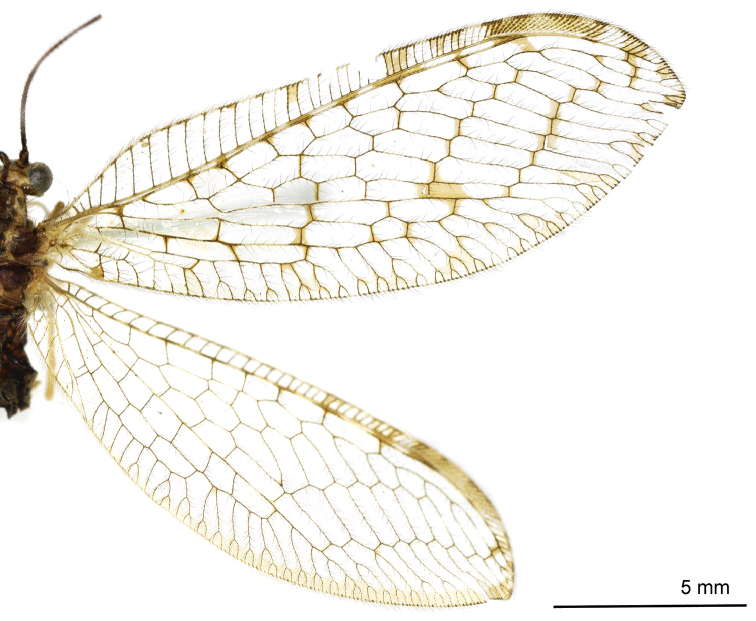
Wings of *Heterosmylus curvagradatus* Yang, 1999.

Body length 7–10 mm. *Head*. Vertex shiny yellow with a brown round spot in middle and a greyish yellow transverse band near antennae; frons bright yellow with a black brown stripe; ocelli grey but black at base; compound eyes grey and glossy; antennae black; clypeus yellow, maxillary and labial palpi dark brown. *Thorax*. Dark brown with two yellow longitudinal stripes from pronotum to mesonotum. mesoscutellum bright yellow, metanotum with a central yellow stripe. *Wings* (Fig. 44). Forewing length 15–17 mm, width 5–6 mm; membrane hyaline, veins fuscous with numerous long setae; pterostigma yellow but brown on both sides. Crossveins *r1-rs* edged with dark brown spots. Rs with 8–9 branches, gradates cross-veins with brown marks. Hind wing length 13–14 mm, width 4–6 mm; membrane hyaline without spots besides the pterostigma.

**Figures 45–50.**
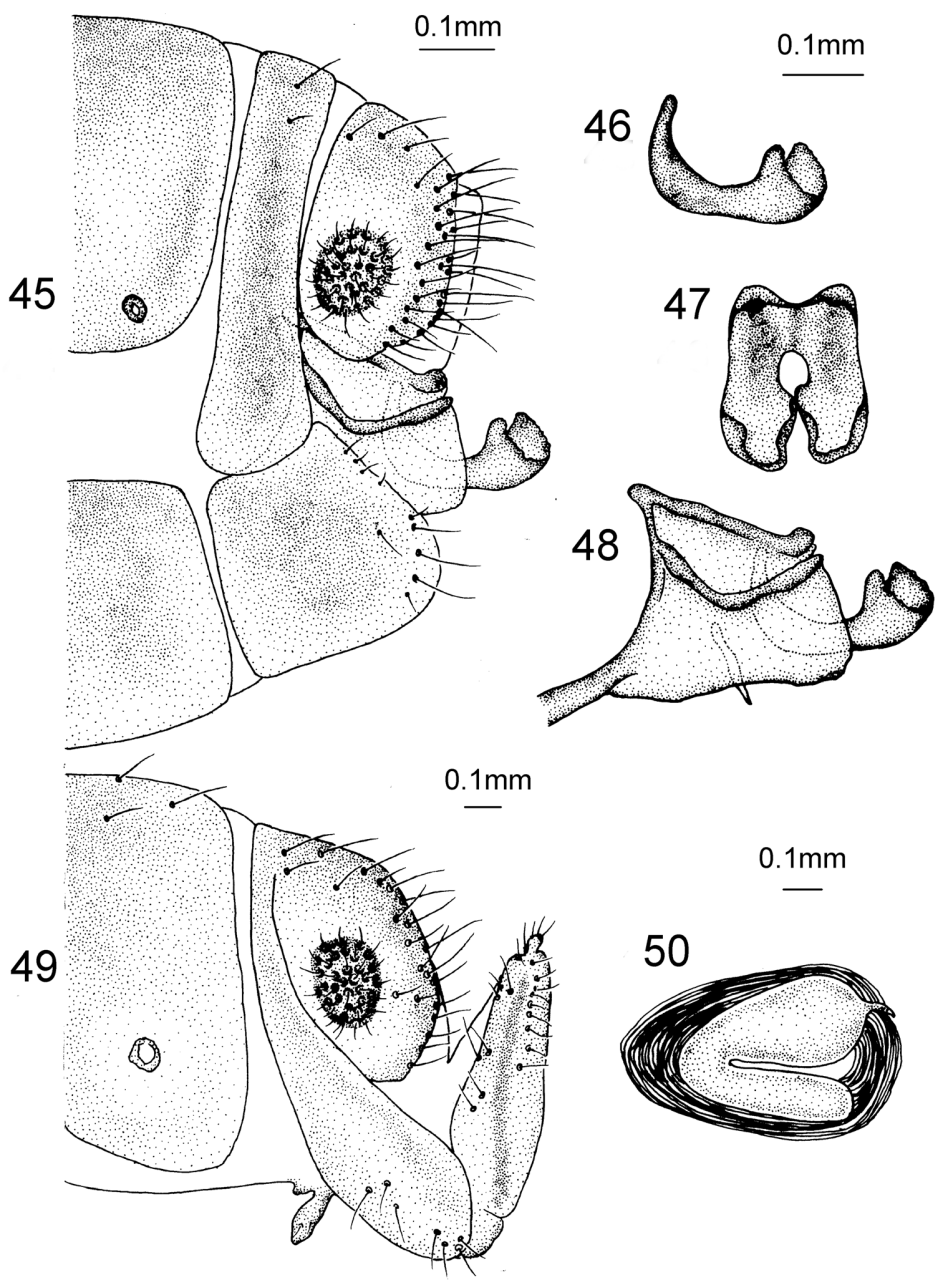
*Heterosmylus curvagradatus* Yang, 1999. Male: **45** terminalia, lateral view **46** mediuncus, lateral view **47** mediuncus, dorsal view **48** genitalia, lateral view. Female: **49** terminalia, lateral view **50** spermatheca, lateral view.

*Male Terminalia* (Figs 45–48). Ectoproct approximately pentagonal in lateral view; gonarcus rod-like in lateral view, apex slightly inflated, short and curved dorsad; mediuncus fused at base and scoop-shaped in dorsal view and apex flat-bottomed in dorsal view.

*Female Terminalia* (Figs 49–50). 8^th^ sternite reduced into finger-shape in lateral view; ectoproct approximately trapezoid in lateral view; 9^th^ gonocoxite approximately finger-shaped in lateral view; 9^th^ gonostylus brown and short; spermathecae bend into C-shape.

#### Distribution.

China (Fujian).

## Supplementary Material

XML Treatment for Heterosmylus

XML Treatment for Heterosmylus processus

XML Treatment for Heterosmylus yunnanus

XML Treatment for Heterosmylus limulus

XML Treatment for Heterosmylus wolonganus

XML Treatment for Heterosmylus flavidus

XML Treatment for Heterosmylus shennonganus

XML Treatment for Heterosmylus curvagradatus
